# The Practice of Dental Medicine

**Published:** 1873-11

**Authors:** E. M. Morrison

**Affiliations:** Des Moines, Iowa


### THE PRACTICE OF DENTAL MEDICINE.

EDITOR DENTAL REGISTER :—

I see in the Transactions of our State Dental Society, a very good address by Dr. Ingersoll, of Keokuk, president of the society, in which he says :—"Local and constitutional treatment for the various diseases of the teeth constitute the distinguishing feature of our times. I therefore call this the "*Therapeutical Era.*"

This may be partially true, but I fear the picture is greatly overdrawn. I have paid much attention to this department of dentistry for a number of years, and I feel that the therapeutical era is quite feeble, and evinces a very superficial knowledge of the principles of medication. I fully concur, however, in the following paragraph, which appeared in the DENTAL REGISTER, for Sept., 1873, p. 401.

"The time is near at hand when the dentist will give far more attention to general hygiene and medication than hitherto, and anything that will tend to bring about such a result, should be received, encouraged and properly used."

We have ever claimed to practice a specialty in medicine, yet most dentists use very little medicine in their practice. It is true, they sometimes apply creosote and arsenic to exposed pulps, but they even do this without any adequate conception of their therapeutical or toxical effects. To come down to the plain truth, a great many claiming rank in our profession do not possess that knowledge which would enable them to treat intelligently the most simple case requiring medication. They are not up to the "*Therapeutical Era*" of Dr. Ingersoll, but he is in the advance guard, and only sees the foreground from the standpoint of his retiring address. This is, perhaps, pardonable under the circumstances, but the status of the profession is not as it should be—up to the standard. We should all understand *special* therapeutics and pathology, and be able to diagnose, prognose and treat the diseases met with in our offices in a legitimate practice ; and be possessed of that general knowledge in these departments, which would enable us to trace the effects of those diseases more remotely affecting the treatment of our patients. So far as I am aware, we have no work devoted especially to the practice of dental medicine, to supply the demands of the profession at the present time. A work of this kind, if properly compiled and arranged, would greatly add to the efficiency of dental practice and instruction, and do more than any other one thing to place our profession in a proper relation to the parent professions. It would require a great deal of labor to produce such a work, as it were, out of chaos, but I hope it will soon be accomplished by some one competent for the undertaking. The leading members of the profession should be called upon in some suitable way, and they should respond cheerfully to aid in this good work, so that when it appears it shall reflect the medical practice and experience of those most competent in our ranks. Let me hear from the REGISTER on this subject.

Yours truly,

DES MOINES, IOWA, E. M. MORRISON.
October 21, 1873.

